# Property database for single-element doping in ZnO obtained by automated first-principles calculations

**DOI:** 10.1038/srep40907

**Published:** 2017-01-23

**Authors:** Kanghoon Yim, Joohee Lee, Dongheon Lee, Miso Lee, Eunae Cho, Hyo Sug Lee, Ho-Hyun Nahm, Seungwu Han

**Affiliations:** 1 Department of Materials Science and Engineering and Research Institute of Advanced Materials, Seoul National University, Seoul 08826, Korea; 2 Platform Technology Lab., SAIT, Samsung Advanced Institute of Technology, 130, Samsung-ro, Yeong Tong-gu, Suwon-si, Gyeonggi-do 16687, Korea; 3 Center for Correlated Electron Systems, Institute for Basic Science (IBS), Seoul 151-747, Korea; 4 Department of Physics and Astronomy, Seoul National University, Seoul 151-747, Korea; 5 Korea Institute for Advanced Study, Seoul 130-722, Korea

## Abstract

Throughout the past decades, doped-ZnO has been widely used in various optical, electrical, magnetic, and energy devices. While almost every element in the Periodic Table was doped in ZnO, the systematic computational study is still limited to a small number of dopants, which may hinder a firm understanding of experimental observations. In this report, we systematically calculate the single-element doping property of ZnO using first-principles calculations. We develop an automation code that enables efficient and reliable high-throughput calculations on thousands of possible dopant configurations. As a result, we obtain formation-energy diagrams for total 61 dopants, ranging from Li to Bi. Furthermore, we evaluate each dopant in terms of n-type/p-type behaviors by identifying the major dopant configurations and calculating carrier concentrations at a specific dopant density. The existence of localized magnetic moment is also examined for spintronic applications. The property database obtained here for doped ZnO will serve as a useful reference in engineering the material property of ZnO through doping.

As a representative semiconducting oxide, zinc oxide (ZnO) is arguably one of the most popular material in science and technology. Its application areas encompass essentially every engineering field; electronic and optoelectronic devices^1^,^2^,^3^, sensors and catalysts^4^,^5^,^6^, and piezoelectric devices for energy harvesting^7^. Such a wide popularity of ZnO stems from several intrinsic/extrinsic advantages as a functional material; low cost, high electron mobility, transparency, and easy fabrication into various nanostructures such as nanorods, nanowires, nanobelts, and nanotubes. Another important advantage of ZnO is facile external doping^8^. As summarized in Fig. 1, the dopants in ZnO cover most of atoms in the Periodic Table, and they vary depending on the application target. Specifically, for transparent electrodes, elements such as Al, Ga, and In (P and As) atoms are incorporated to increase electron (hole) carrier densities^9^,^10^,^11^,^12^. To enhance absorption of visible lights, various dopants are chosen that create defect levels within the band gap or shift band-edge positions, narrowing the optical gap^13^,^14^. In sensor and catalyst applications, transition and novel metals are doped to increase active sites that accelerate chemical reactions^5^,^6^. Lastly, the magnetic impurities are incorporated to use ZnO as the spin-transport layer in spintronic devices^15^.

Above examples show that doping is a powerful tool to tailor the optical, electrical, chemical, and magnetic properties of ZnO. However, the detailed mechanism underlying the property tuning is not easily revealed by experimental analysis alone because defects or dopants are difficult to investigate with spectroscopic tools. Theoretical study based on the first-principles calculations is very useful in this respect, and several studies successfully enlightened the atomistic origin of property changes induced by dopants^16^,^17^. However, systematic and thorough studies have been limited to representative elements^12^,^16^,^18^,^19^,^20^,^21^,^22^,^23^,^24^,^25^,^26^. In Fig. 1, elements that have been fully studied are marked in red outlines while those in blue indicate partial studies. Here, the ‘fully studied’ means that i) possible dopant configurations have been compared with respect to the defect formation energy and ii) experimental conditions such as the oxygen pressure are taken into account. If any of these considerations is missing, it is tagged as ‘partial’. In addition, many studies were carried out within semilocal functionals such as the local density approximation (LDA) or the generalized gradient approximation (GGA), often with the addition of the on-site Hubbard term^27^. However, the band-gap underestimation by these functionals is particularly acute in ZnO^28^ and several literature corroborates that the band-gap correction is essential in defect energetics in ZnO^29^,^30^.

Above discussions indicate that it is timely to compile a full table of dopant-related property change for every possible dopant in ZnO, which cover essentially all the elements in the Periodic Table. To compare various dopant configurations for so many dopants, thousands of supercell structures should be calculated, which would be formidable if one tries to carry out computations in a manual way. In a previous work, we demonstrated high-throughput screening of high-k oxides by overcoming difficulties in massive calculations through the reliable automation procedure^31^. Similarly, in order to establish extensive property databases of doped-ZnO in a consistent and systematic way, we develop in this work a first-principles-based automation code that explores various dopant configurations and yields formation-energy profiles for any given dopant. Using the automation code, we conduct high-throughput calculations for 61 dopants from the Periodic Table, ranging from Li to Bi, and compile property databases and formation energy diagrams. For lanthanides, we calculate La as the representative case since all the members in the series share similar chemical properties. We also examine the possibility of each dopants for n-type/p-type semiconductors and magnetic applications.

## Results

### Automation workflow

The schematic work flow of the automation code is depicted in Fig. 2. More details are provided in the Methods section. Once an element is selected, the code generates possible initial dopant geometries that consist of substitutional, void-interstitial, and split-interstitial configurations. Then, all the configurations with various charge states are relaxed within the GGA + *U* functional. As results, 20~100 relaxed configurations and their formation energies are obtained for each element. As mentioned above, the GGA + *U* functional significantly underestimate the band gap which affects the formation energy. To address this issue in a high-throughput style, we first screen stable dopant configurations with low formation energies on the basis of GGA + *U* results. Then, we apply one-shot hybrid functional calculation by fixing atomic positions to the GGA + *U* structure. The errors from this approximation are mostly small (≤0.2 eV; see the Methods section). The exception is when the nature of defect state is different between GGA + *U* and hybrid functionals. For instance, in Li_Zn_ and Na_Zn_, the distribution of defect state is much more localized in the hybrid functional calculations than in GGA + *U*. In such cases, we carry out the full relaxation within the hybrid functional.

### Formation energy diagrams

We calculate the formation energy of each configuration (*E*_for_) using the following equation:


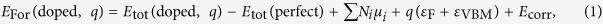


where *q* is the charge state, *N*_*i*_ and *μ*_*i*_ are the number and chemical potential of the chemical species *i (i* = Zn, O, and dopant). In equation (1), *ε*_F_ is the Fermi energy with respect to the valence band maximum energy (*ε*_VBM_), and is *E*_corr_ the correction energy for the finite supercell. The chemical potentials can vary depending on the thermodynamic environment and we consider O-rich and Zn-rich conditions as two opposing limits. For the dopant chemical potential, we calculated all the unary and binary (Zn-dopant and dopant-O) phases from ICSD, and selected the minimum chemical potential among them. This also means that the dopant chemical potential can assume different values in Zn-rich and O-rich conditions.

**Figure 1:**
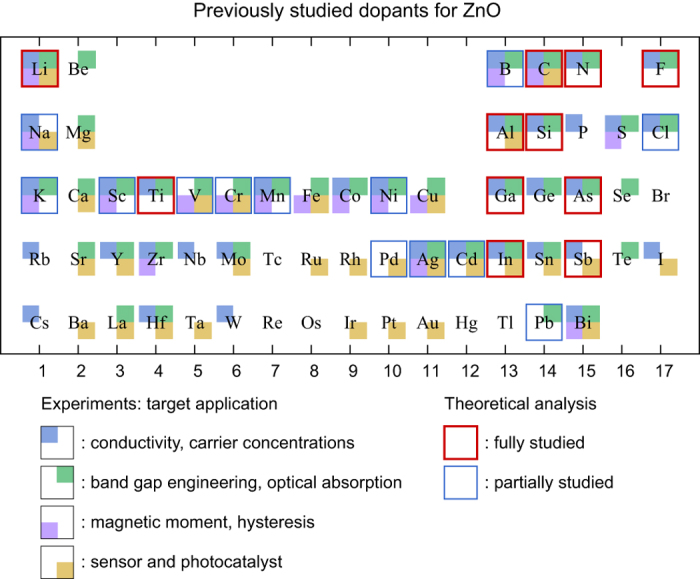
Previously studied dopants for ZnO that are collected from ~550 publications. Each target application in experiment is color coded in small squares. Theoretically studied dopants are indicated by border lines with a color distinguishing fully and partially studied works. Although not shown here, most elements in lanthanides were also doped to activate luminescence from intra 4*f*-transitions^70^.

**Figure 2:**
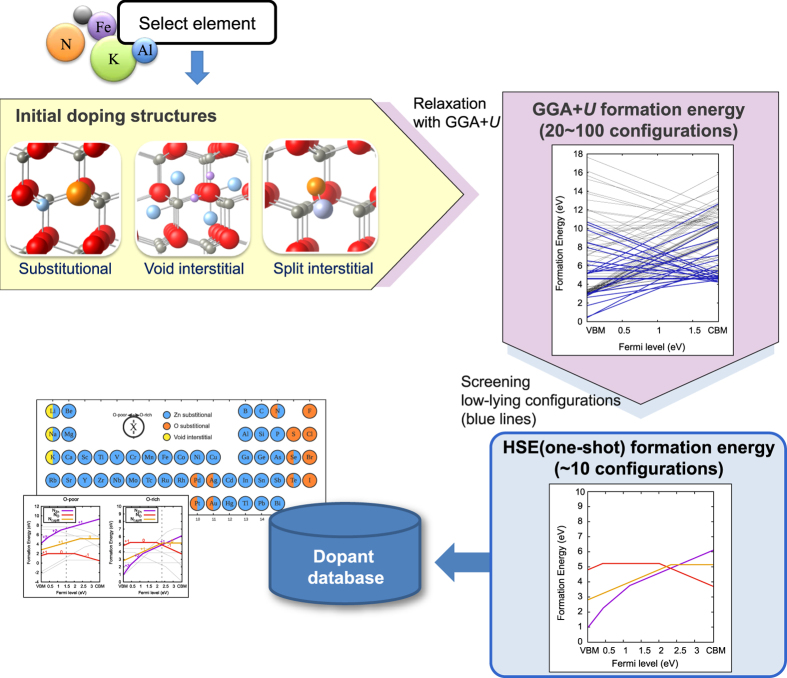
Schematic workflow in automation code for high-throughput calculations of doped ZnO.

**Figure 3:**
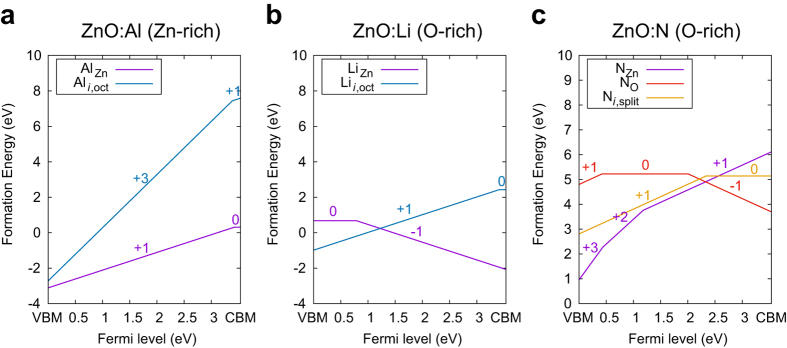
Formation energy plots of representative dopants. (**a**) Al, (**b**) Li, and (**c**) N.

In the Supplementary Information, all the formation energy plots of the 61 dopants in Fig. 1 are compiled (see Supplementary Fig. S1; structure files are also provided in Supplementary Dataset). For completeness, we also provide the result for hydrogen in Fig. S2. For the validity check, we present in Fig. 3 results for three well-known dopants; Al, Li and N. In Fig. 3a, the Al dopant substituting Zn becomes a shallow donor because the (+1/0) transition level lies just below the conduction minimum, which is consistent with other literatures^16^,^32^,^33^. Figure 2b for Li-doped ZnO shows that acceptors by 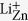 and donors by 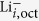 (interstitial Li at the octahedral site) are self-compensating as known in former studies^18^,^34^. The calculated (0/−) thermal transition level of Li_Zn_ is 0.79 eV above VBM, in good agreement with experimental estimation of 0.8 eV^35^ and previous hybrid calculation^34^. In the case of N-doped ZnO, shown in Fig. 3c, N_O_ is a deep acceptor with the (0/−1) transition level of 2.0 eV that is close to 2.1 eV in the former theoretical study^36^. Interestingly, we find that N_Zn_ is stable over a wide range of the Fermi level in the O-rich condition although N_Zn_ has never been considered in the previous studies as far as we are aware. The local relaxation indicates that the N atom replacing Zn forms bonds with three neighboring O atoms, reminiscent of the NO_3_ molecule. Thus, the results on Al, Li, and N dopants confirm that the automated procedure can produce formation energies reliably.

### Major dopant configurations

To investigate the influence of dopants on the electronic property of ZnO, the dopant concentration should be determined first. Under the ideal equilibrium condition, the dopant concentration varies within the solubility limit. However, many experimental data indicate that thin-films of ZnO often grow through non-equilibrium processes^37^,^38^ such that dopants can be incorporated much beyond the equilibrium solubility limit^38^,^39^,^40^. To reflect this and also for computational convenience, we fix the total dopant concentration to 10^20^ cm^−3^ (~0.2 at.%) and determine the relative population between different configurations according to the ratio of Boltzmann factors.

Considering all dopant concentrations together with intrinsic defects, one can determine the Fermi level at a certain temperature by imposing the charge neutrality condition:


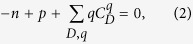


where *n* and *p* are electron and hole concentrations, respectively, and 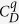 is the concentration of a defect or dopant type *D* with the charge state of *q*. We include the formation energies of intrinsic defects such as *V*_O_, *V*_Zn_, O_*i*_, Zn_*i*_, and O_*i*,split_ in equation (2). Unlike the dopant whose total concentration is fixed to 10^20^ cm^−3^, the density of intrinsic defects follows the equilibrium distribution given by the Boltzmann factor.

**Figure 4:**
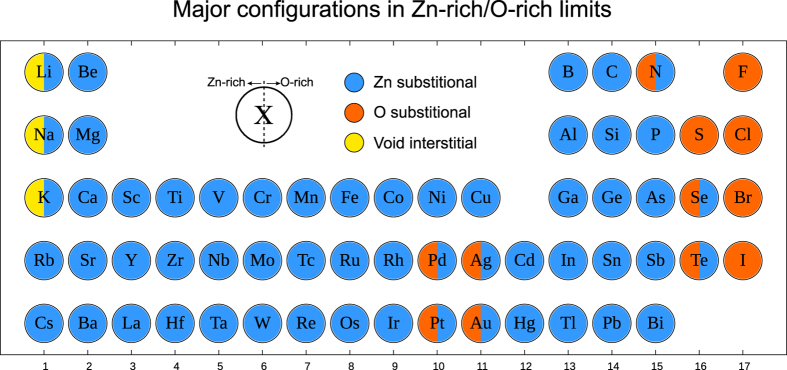
Major dopant configurations in Zn-rich (left half-circle) and O-rich (right half-circle) conditions. Dopant concentrations are fixed to 10^20^ cm^−3^ and the temperature is 300 K.

Figure 4 presents the major dopant configurations at 300 K in Zn-rich and O-rich conditions. It is seen that Zn substitutional dopants are dominant for most metals in both conditions, while small alkali metal dopants (Li, Na, and K) prefer interstitial sites in Zn-rich conditions. The O substitutional doping is stable for several semi-metal and non-metal elements in Zn-rich condition while halogen dopants are always stable at the O substitutional site. No split-interstitial configuration is identified as the major configuration for studied elements. On the other hand, it is interesting that Pd, Ag, Pt and Au dopants are stable when they replace oxygen in Zn-rich condition. By inspecting the electronic structure, we find that these group 10 and 11 transition metals are chemically stable at oxygen sites as they accept two electrons from neighboring Zn ions and so their *d*^10^ states are completely filled. Such counter-intuitive configurations would be difficult to consider without the automation procedure employed in this work.

### Carrier concentrations

**Figure 5:**
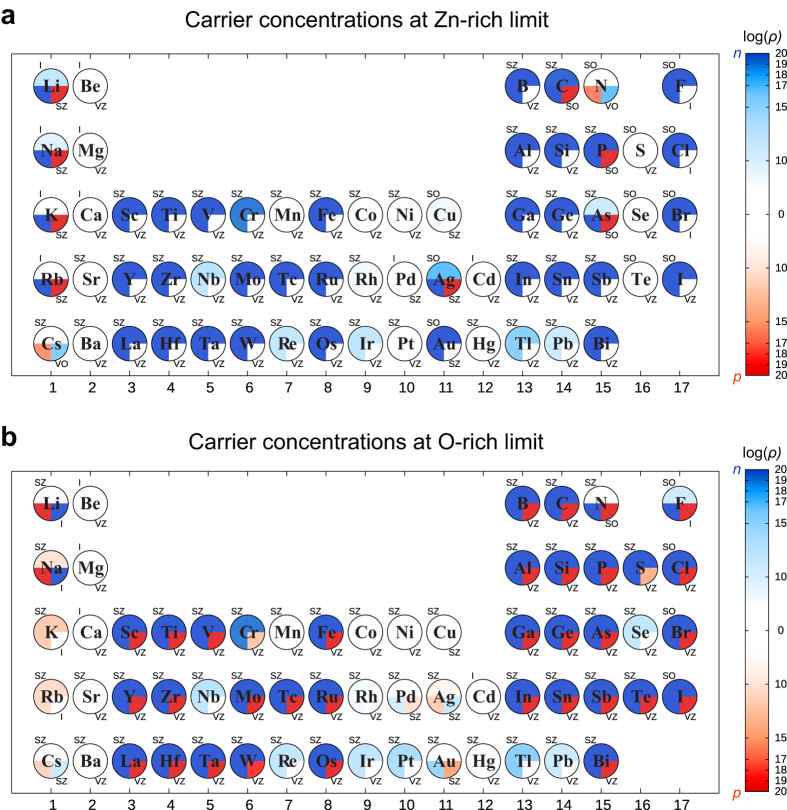
Carrier concentrations at (**a**) Zn-rich and (**b**) O-rich conditions when the dopant concentration is fixed to 10^20^ cm^−3^ at 300 K. The blue and red represent the electron- and hole-carrier concentration, *n* and *p*, respectively. The total carrier concentration is color-coded within the upper half circle. The lower-left quadrant indicates the concentration of major charged configuration and the lower-right quadrant indicates the density of its largest compensating defect. The dopant or defect concentrations are colored according to their charge state (blue (red) indicates + (−) charge state and donor (acceptor)-like nature). Labels around the circle indicate the type of major or compensating defects (SZ: Zn substitutional, SO: O substitutional, I: interstitial, VZ: Zn vacancy, VO: oxygen vacancy).

In Fig. 5, we present the calculated total carrier concentrations at 300 K that result when the dopant concentration is 10^20^ cm^−3^. The total carrier concentration (upper hemisphere), major charged dopant concentration (lower left quadrant), and its compensating defect concentration (lower right quadrant) of each dopant are color-coded within each circle. The corresponding major and compensating defect types are indicated outside the circle. From the color map, one can easily capture the electrical property of dopants in the limit conditions and understand how each dopant contribute to the carrier concentration. For example, F-doped ZnO in the Zn-rich condition is n-type with the electron concentration of ~10^19^ cm^−3^ due to the formation of electron-donating F_O_ and the low density of compensating F_*i*_. In contrast, in O-rich condition, the same F_O_ is largely compensated by F_*i*_ that plays as an acceptor, resulting in the low electron carriers (~10^7^ cm^−3^).

### Local magnetic moment

**Figure 6:**
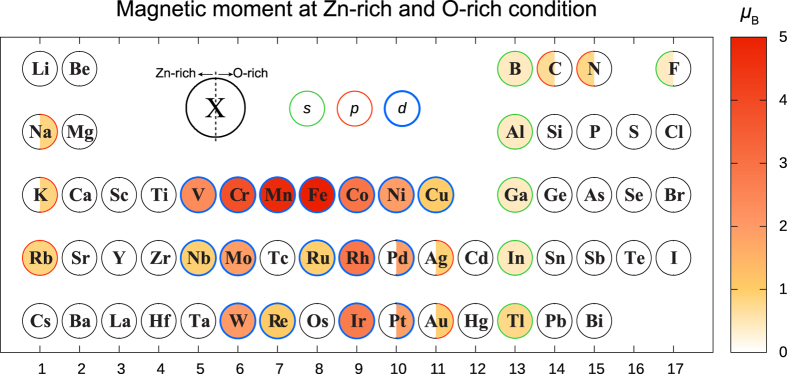
Magnetic moments of major dopant configuration in Bohr magnetron (*μ*_B_). The color of border lines indicates the orbital symmetry of the polarized spin density.

Certain dopants can have unpaired electrons with finite magnetic moments in their stable defect configurations. If the magnetic moments are localized on the dopants, it can be utilized as diluted magnetic semiconductors. In Fig. 6, we plot the net magnetization of the major dopant configurations at Zn-rich and O-rich limit conditions. The net magnetic moments are obtained by integrating the difference between spin-up and spin-down densities. The orbital symmetry that mainly contribute to the magnetic moment is marked by the border color. (The decomposition was done by using the PAW projector.) When the *d*-orbital is the major contributor, the magnetization is always strongly localized at the dopant ion. In the case of *s*- and *p*-orbital symmetries, on the other hand, the spin moment is distributed throughout the supercell and so they will not form a localized moment that is necessary for magnetic applications.

## Discussion

As mentioned above, n-type doping is prevalent for many dopants in both Zn-rich and O-rich conditions. Elements in groups 3, 13, 14, and 17 are already well-known as n-type dopants. In particular, the group 13 elements such as B, Al, Ga and In are preferred dopants in actual applications^9^,^10^. Rare-earth dopants in group 3 are comparable to group 13 dopants in the present result as well as in the experimental study^41^. Elements in group 14 are also good electron-producers as they donate two electrons per dopant to the conduction band. Unlike other n-type dopants, group 17 halogen elements donate electrons by substituting oxygen, as they need only one electron for satisfying the octet rule. Since F^−^ ion has a similar size with O^2−^ ion, the calculated formation energy of 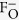 is the lowest among halogen elements. This is consistent with low resistivity of F-doped ZnO (~10^−4^ Ω∙cm)^42^ that is comparable to Al- and Ga-doped ZnO. Cl is also known as a good n-type dopant in experiment^43^. On the other hand, I is expected to be an electron donor from Fig. 5, which is at variance with experiment^44^. This is due to the low dopability of I, which result in precipitates at high doping concentrations^44^. This is in fact in line with the current calculations because *E*_for_ of I is bigger than for F by ~2 eV (see Supplementary Fig. S1). Although Br-doped ZnO has not been studied as far as we are aware, it would behave similarly to I considering its high *E*_for_.

In Fig. 5, many transition metals in groups 4–9 can make ZnO good n-type semiconductors, because they mostly occupy the Zn site and their valence numbers are larger than for Zn (+2). Dopants such as Mn and Ni do not produce electron carriers because the valence state of +2 is stable for them. *E*_for_’s among group 4–9 dopants lies between 0~4 eV. Considering that several transition-metal elements (Ti, Zr, Hf, Cr, and Mn) can be doped in ZnO^14^,^23^,^45^,^46^, we believe that most dopants in these groups could be incorporated into ZnO. We note that V, Tc, Ru, La, Ta and Os atoms have not been tested as n-type dopants as far as we are aware. It is also noticeable that the chalcogen atoms such as S, Se, and Te are identified as n-type dopants under the O-rich condition. In this condition, the chalcogen substitutes Zn, rather than O, and hence donates electrons. Indeed, n-type behavior was observed for S-doped ZnO with resistivity of ~0.5 Ω∙cm^47^. Among other parts of the Periodic Table, Au is noted as the n-type dopant in the Zn-rich condition. The Au dopant donates electrons by substituting the oxygen atom as mentioned in the previous section. The carrier concentration in Au-doped ZnO decreases with oxygen pressure as the dopant site shifts from Au_O_ to Au_Zn_ that is a deep donor.

From Fig. 5, we could not identify any noticeable p-type dopants. Several dopants show p-type behaviors, but their carrier concentrations are too low (<10^10^ cm^−3^) for practical applications. Intuitively, group 1 and 15 dopants would behave as acceptors under O-rich conditions by substituting Zn and O, respectively. For alkali metals, however, the donor states in the Zn substitution are always deep. Among group 15 elements, N is the most extensively studied dopant to synthesize p-type ZnO, although theoretical studies commonly suggested that N_O_ is the deep acceptor^23^,^48^. This is also confirmed by the present work as the transition level *ε*(0/−1) is computed to be ~2 eV from VBM (see Fig. 3(c)). Except for N, other group 15 elements favor n-type Zn substitution, rather than O substitution, which is consistent with other theoretical studies^12^,^26^. The difficulty of p-type doping in ZnO can be understood in terms of localization and energy alignment of valence states; the valence states of ZnO is composed by highly localized O-*p* orbitals and the ionization potential is very large (~7.4 eV) implying large energy costs for inducing hole carriers via defect^49^. The native and hydrogen-related defects reflect this aspect (see Supplementary Figs S2 and S3) —the intrinsic acceptors *V*_Zn_^2−^ and O_*i*_^2−^ both have deep nature and relatively high formation energies compared to intrinsic donors (*V*_O_^2+^ and Zn_*i*_^2+^). In the case of hydrogen interstitial, H_*i*_^+^ is a shallow donor as the acceptor state is unstable. (As *V*_O_^2+^ and Zn_*i*_^2+^ are deep donors, H_*i*_^+^ is considered as the source of unintended n-type conductivity in ZnO^50^). In experiment, however, p-type could be obtained for N-, As- and Sb-doped ZnO^11^,^51^,^52^ and both n- and p-type behaviors were reported for P-doped ZnO depending on the dopant concentration^53^. The mechanism of hole production was explained by defect complexes^12^,^26^,^52^, which is beyond the scope of the present work.

For magnetic applications, dopants with large magnetic moments in Fig. 6 are mostly 3*d*-transition metals (V, Cr, Mn, Fe, Co, and Ni) that were extensively studied as diluted magnetic semiconductors^45^,^54^,^55^,^56^,^57^,^58^. We expect that Mo, Rh, W, and Ir can be new candidates for magnetic applications as they exhibit substantial magnetic moments of 2~3 *μ*_B_. For these dopants, the major configuration (Zn substitution) does not change from Zn-rich to O-rich conditions. In the O-rich condition, Pd and Pt also have sizeable magnetic moments of ~2 *μ*_B_. In passing, we note that rare-earth atoms such as Nd and Gd were also studied for room-temperature ferromagnetism^59^ because they possess large magnetic moments from the localized *f* electrons.

In summary, we established an automated first-principles procedure to build a comprehensive single-dopant property database for ZnO. Through the automation code, we computed thousands of dopant configurations in consistent and reliable ways, thereby assembling the whole formation energy profiles for 61 dopants in the Periodic Table. Examining several representative dopants (Li, Al and N), we confirmed that the results from the automated computations are consistent with previous theoretical studies. In addition, we could identify several dopant configurations that are stable but have not been studied theoretically to date. Assuming the constant dopant concentration of 10^20^ cm^−3^, we investigated the major dopant configurations and total carrier concentrations at 300 K, and suggested new candidate dopants for n-type and spintronic applications. By establishing a property database for every possible dopant in the Periodic Table, we believe that the present work can contribute to engineering the material property of ZnO through doping. In addition, the computational procedure established here could be applied to compiling a massive database for dopants in other materials.

## Methods

### Computational details

Vienna Ab initio Simulation Package (VASP)^60^ is adopted as the core engine of the present first principles calculations based on the density functional theory (DFT). We employ the generalized gradient approximation (GGA)^61^ for the exchange-correlation functional between electrons. The position of Zn-*d* level is significantly overestimated in the conventional DFT calculation, which can be resolved by GGA + *U* calculations. We use the effective on-site interaction energy of *U*−*J* = 7.5 eV on Zn 3*d* electrons, which gives overall agreement of the band structure and density of states with experiments^62^. We also applied the on-site Hubbard interaction to other 3*d*-transition metal dopants with parameters fitted to reproduce experimental formation enthalpies of transition metal oxides^63^. The energy cutoff of the plane-wave basis is set to 400 eV. The 4 × 4 × 3 Monkhorst-Pack **k**-point sampling is used for the crystalline wurtzite ZnO without dopants. For dopant calculations, we use 128-atom (4 × 4 × 2) supercell and Γ-point sampling. For the hybrid calculation, we used the HSE06 functional^64^. The fraction of Fock exchange is set to 0.37 that adjusts the calculated band gap to the experimental value of 3.4 eV. We do not perform ionic relaxations in the hybrid calculation except for Li_Zn_ and Na_Zn_ (see below), and the calculated band gap of one-shot HSE06 on GGA + *U* structure is 3.54 eV. Spin-polarization is considered in every calculation of dopant configurations and intrinsic defects. The chemical potentials of Zn, O, and dopant are determined by the total energies of Zn(hcp), O_2_ molecule, unary phase, and binary phases formed between the dopant and Zn or O. In the O-rich condition, *μ*_O_ is equal to ½*E*_tot_(O_2_) while the Zn-rich condition equalize *μ*_Zn_ with *E*_tot_(Zn(hcp)). The relation *μ*_O_ + *μ*_Zn_ = *E*_tot_(ZnO) is always imposed.

### Screening dopant configurations

#### Initial configurations

To search the stable dopant configurations, we consider all kinds of possible configurations as initial structures. (See Supplementary Fig. S4) First, the cation and anion substitutions are considered. For interstitial dopants, we distinguish void and split configurations. The void interstitial means the interstitial dopants at void sites in the wurzite structure. We use the bonding and anti-bonding model to search the void interstitial sites^65^. The initial distance between the dopant and the nearest atom in void interstitial is set to the sum of ionic radii of dopants and host atoms. The split interstitial corresponds to dumbbell structures at the oxygen site. Regarding the angle of the dumbbell, we found from extensive tests that the split interstitial always prefers high-symmetry directions in the tetrahedron formed by neighboring Zn atoms. (See Supplementary Fig. S4c) Therefore, we consider only these directions in high-throughput computations. Since the split interstitial and void interstitial at the bonding site are feasible for small ions, we first computed on those configurations for smaller elements. We also take into account various charge states on the basis of chemical nature of each element. (See Supplementary Fig. S5) Overall, 20~100 initial configurations (~34 on average) are computed per element.

#### Relaxation

We first relax initial dopant configurations using GGA + *U* functional until atomic forces are reduced to within 0.02 eV/Å. For the interstitial dopant configuration, the initial structures often entail large atomic forces on dopant and neighboring atoms. In these cases, the conventional relaxation method can result in metastable structures or unintended defect complexes. To avoid this and obtain the target dopant configuration, we adopt a two-step process. In the first step, we fix the dopant and atoms in bulk-like region (five atoms furthest from the dopant) and carry out 10 steps of conventional ionic relaxations, which reduces atomic forces significantly while maintaining the interstitial configuration. Then, all atoms are fully relaxed.

#### Hybrid functional calculations

To reduce the computational cost and enable high-throughput computations, we apply the hybrid functional method only for ~10 low-lying dopant configurations from GGA + *U* results. To be specific, we first identify 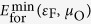, the minimum *E*_for_ at a certain Fermi level (*ε*_F_) and the chemical potential of oxygen (*μ*_O_), among considered dopant configurations. If a certain dopant type has *E*_for_ within 4 eV from 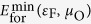 at any *ε*_F_ and *μ*_O_ in the considered ranges, it is passed for the hybrid calculation. To further reduce the computational cost, we applied the one-shot hybrid calculation on the geometries relaxed within GGA + *U*, assuming the relaxed geometries are similar among the two functionals. To check this, we carried out full relaxations for N_O_ and Hf_Zn_ within the hybrid functional and found that the relaxation reduces the energy less than 0.2 eV. (See Supplementary Fig. S6) For the first-row alkali atoms, in particular Li and Na, it is known that GGA + *U* fails to describe the hole polaron in the Zn-substitutional site^31^. Thus, we carried out the full relaxation on Li_Zn_ and Na_Zn_.

### Cell-size corrections

Since we use the supercell approach to calculate the defect formation energy, it is necessary to remove spurious electrostatic interactions among image charges in the repeated cells. We applied the monopole correction^66^ together with the potential alignment^67^. The band-filling correction^68^ was omitted because we sampled only the Γ point. It is known that the neutral defect also suffers from significant cell-size effects if the defect state is delocalized^69^. Therefore, we applied the monopole correction in such cases. (See Supplementary Fig. S7 for Hf_Zn_^0^.) We tested several dopant configurations and confirmed that the Γ-point provides well-converged results with respect to the **k**-point sampling. (For instance, see Fig. S7b.)

### Magnetic moments

The net magnetic moments in Fig. 6 are obtained by integrating the difference between spin-up and spin-down densities. The decomposition into *s*-, *p*-, and *d*-orbitals was done by using the PAW projector.

## Additional Information

**How to cite this article**: Yim, K. *et al*. Property database for single-element doping in ZnO obtained by automated first-principles calculations. *Sci. Rep.* **7**, 40907; doi: 10.1038/srep40907 (2017).

**Publisher's note:** Springer Nature remains neutral with regard to jurisdictional claims in published maps and institutional affiliations.

## Supplementary Material

Supplementary Information

Supplementary Dataset 1
