# Supplementary material for: Property database for single-element doping in ZnO obtained by automated first-principles calculations
Source: Sci Rep. 2017 Jan 23;7:40907. doi: 10.1038/srep40907 (PMC5256097; doi:10.1038/srep40907)
Supplement: Supplementary Information [file srep40907-s1.pdf]

Property database for single-element doping in ZnO obtained by automated first-principles calculations

Kanghoon Yim[1], Joohee Lee[1], Dongheon Lee[1], Miso Lee[1], Eunae Cho[2], Hyo Sug Lee[2], Ho-Hyun Nahm[3,4] & Seungwu Han[1,5]*

[1]Department of Materials Science and Engineering and Research Institute of Advanced Materials, Seoul National University, Seoul 08826, Korea

[2]Platform Technology Lab., SAIT, Samsung Advanced Institute of Technology, 130, Samsung-ro ,Yeong Tong-gu, Suwon-si, Gyeonggi-do 16687, Korea

[3]Center for Correlated Electron Systems, Institute for Basic Science (IBS), Seoul 151-747, Korea

[4]Department of Physics and Astronomy, Seoul National University, Seoul 151-747, Korea

[5]Korea Institute for Advanced Study, Seoul 130-722, Korea

Supplementary information

Group 1 elements (IA)

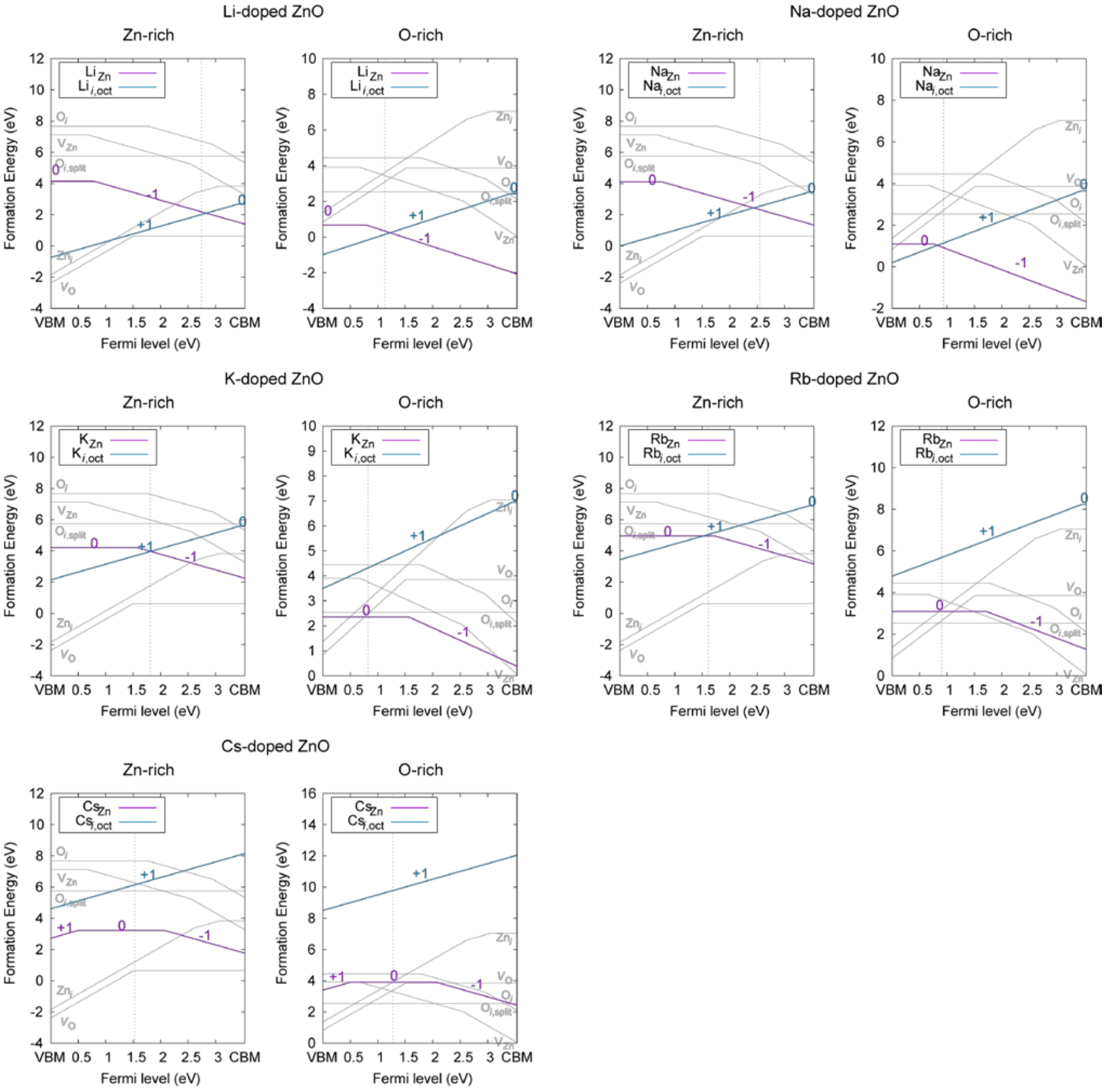

Group 2 elements (IIA)

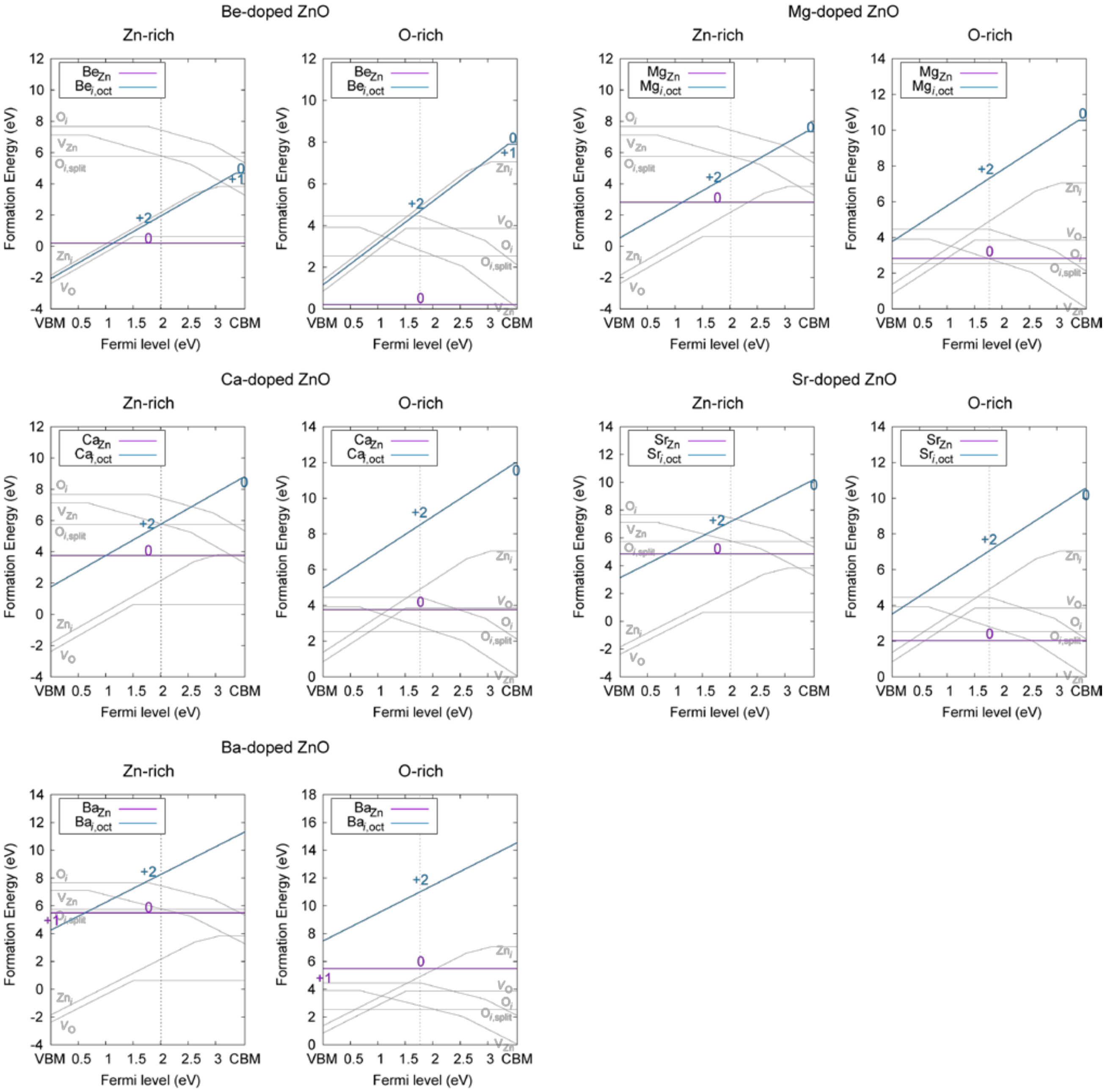

Group 3 elements (IIIB)

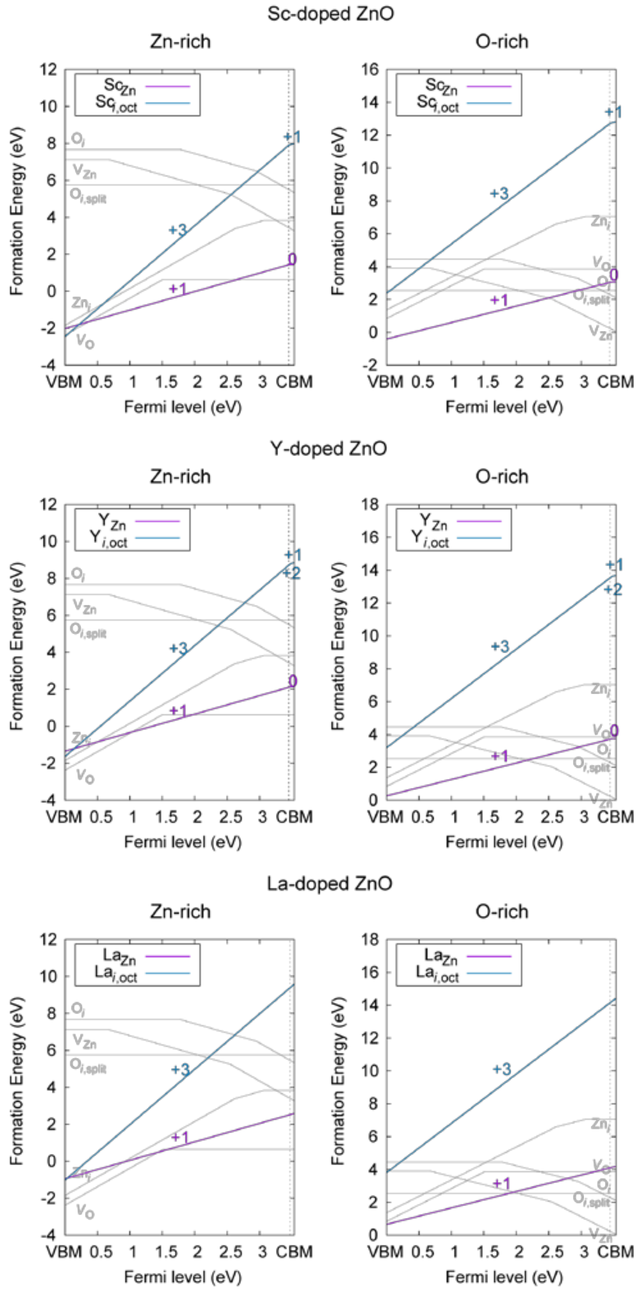

Group 4 elements (IVB)

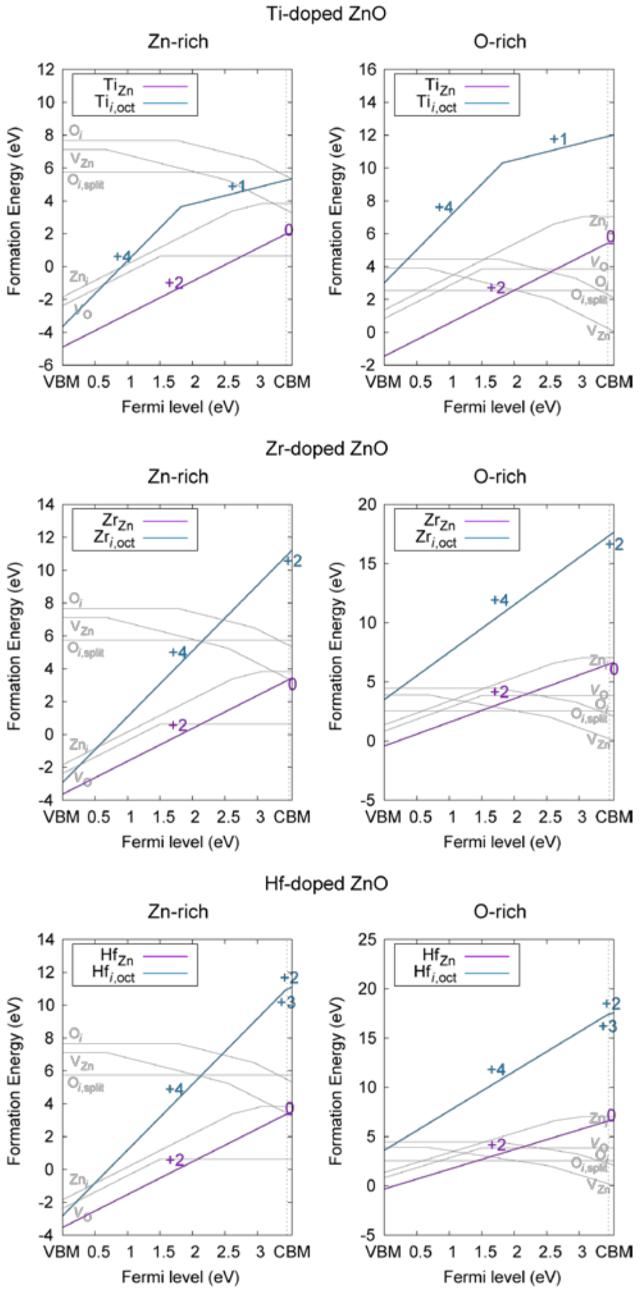

Group 5 elements (VB)

Group 6 elements (VIB)

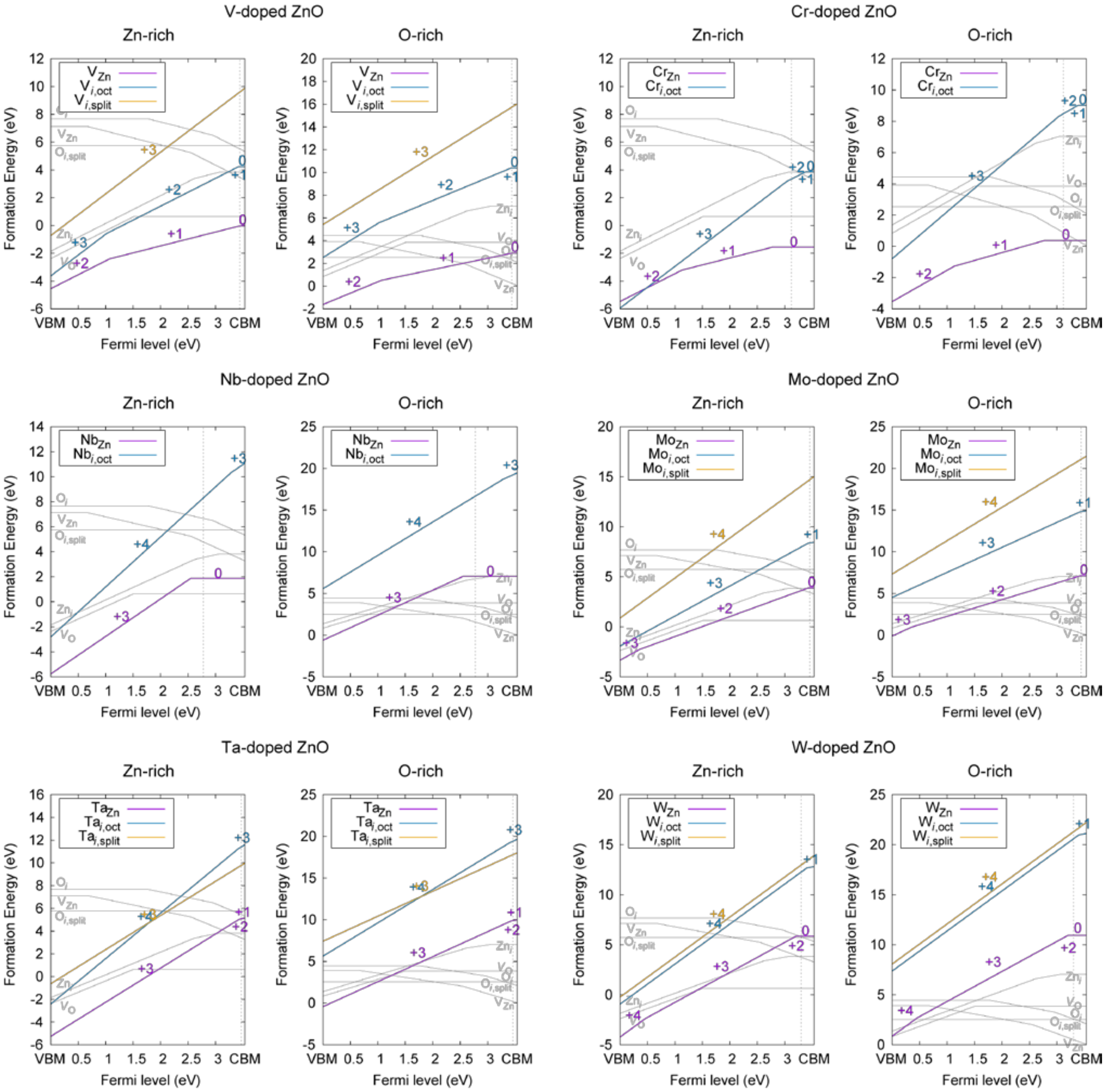

Group 7 elements (VIIB)

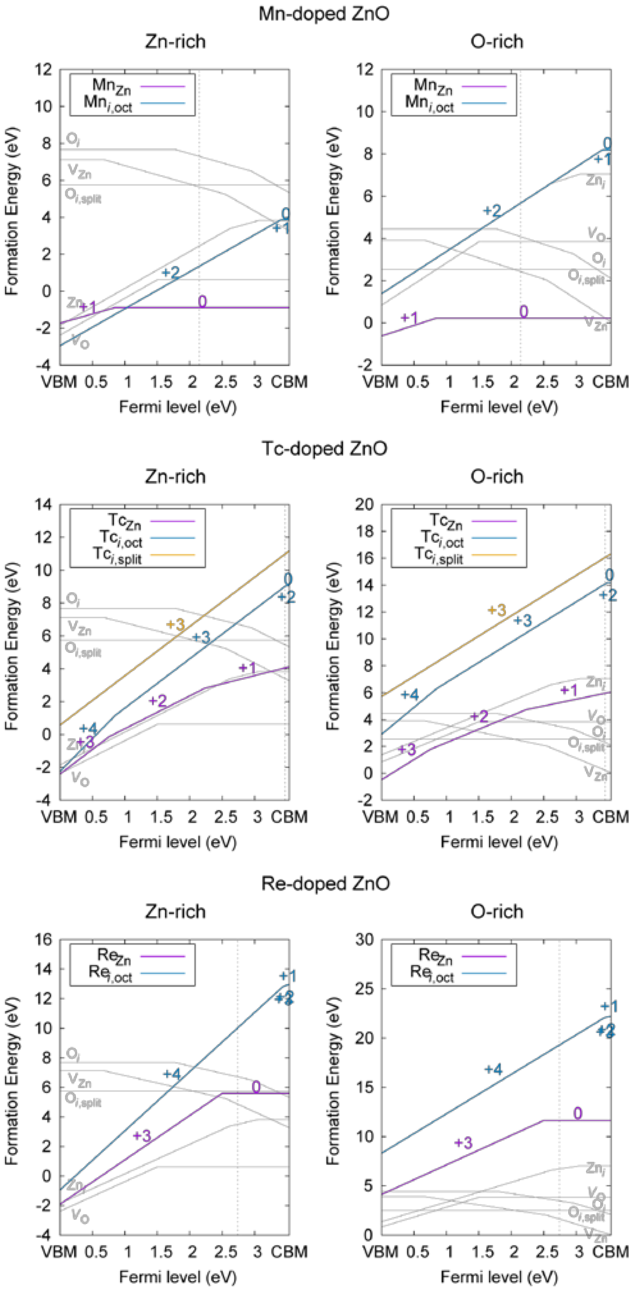

Group 8 elements (VIII)

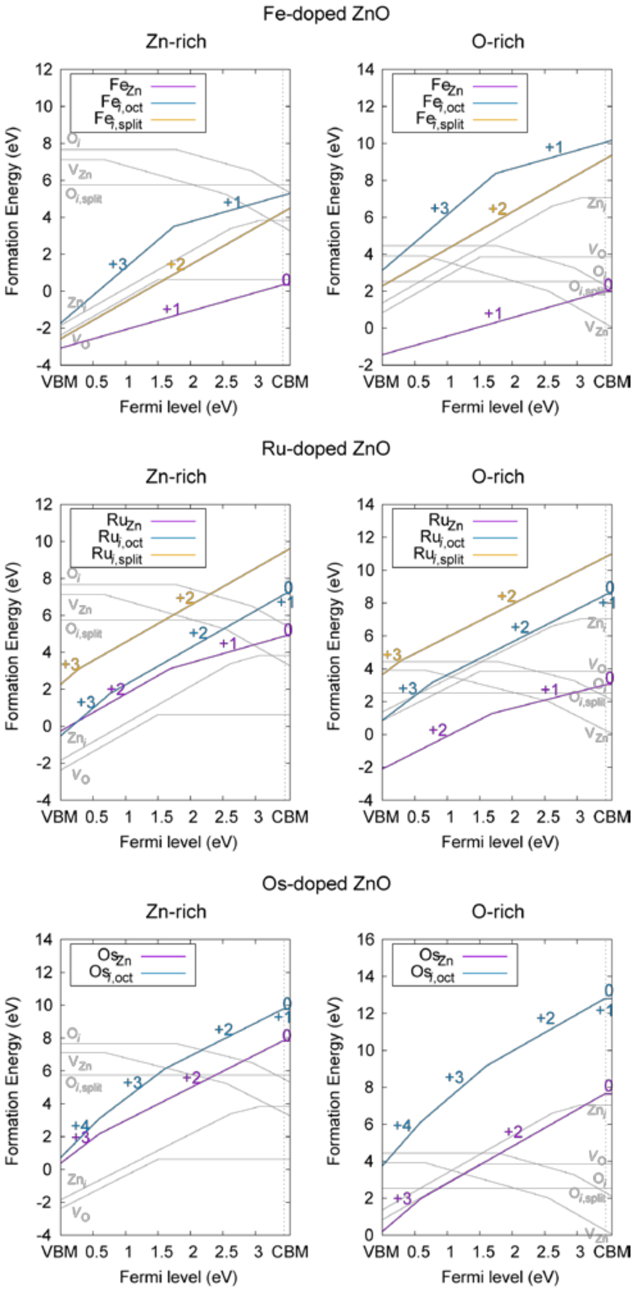

Group 9 elements (VIII)

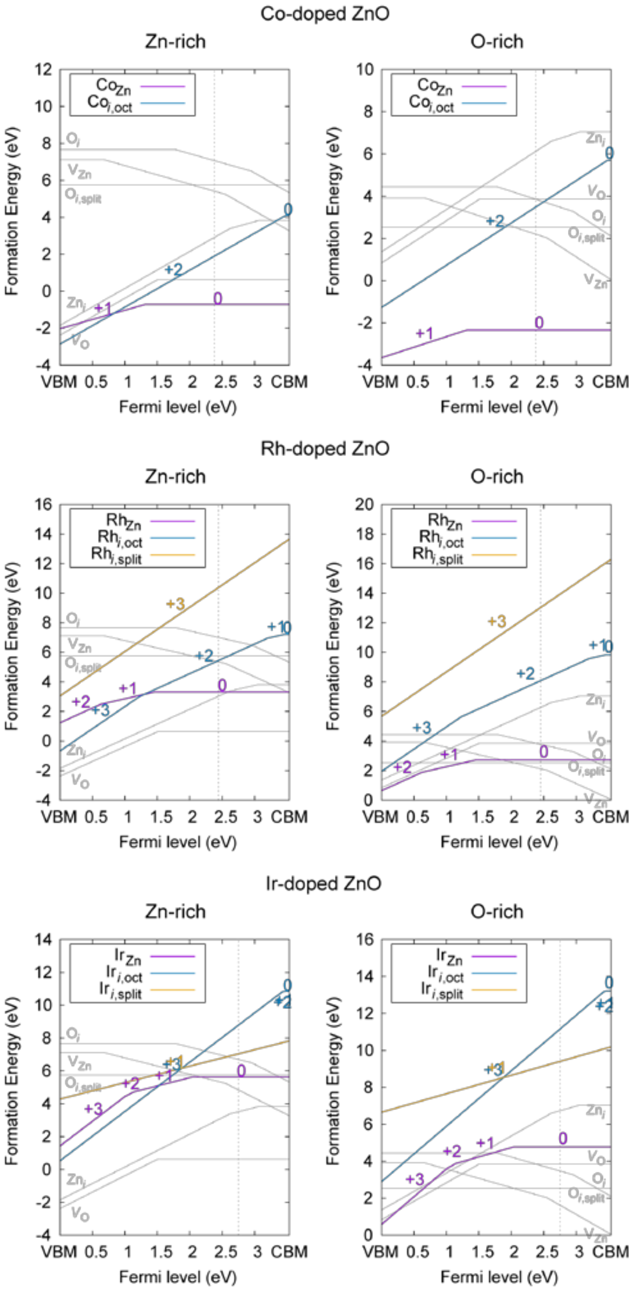

Group 10 elements (VIII)

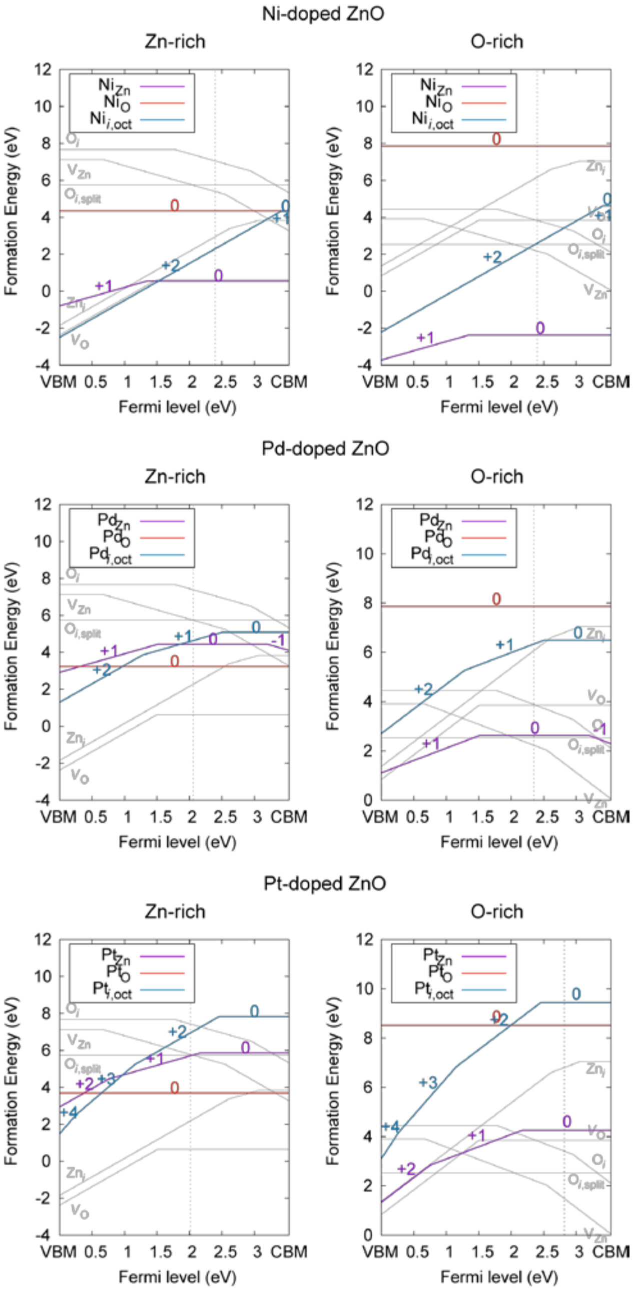

Group 11 elements (IB)

Group 12 elements (IIB)

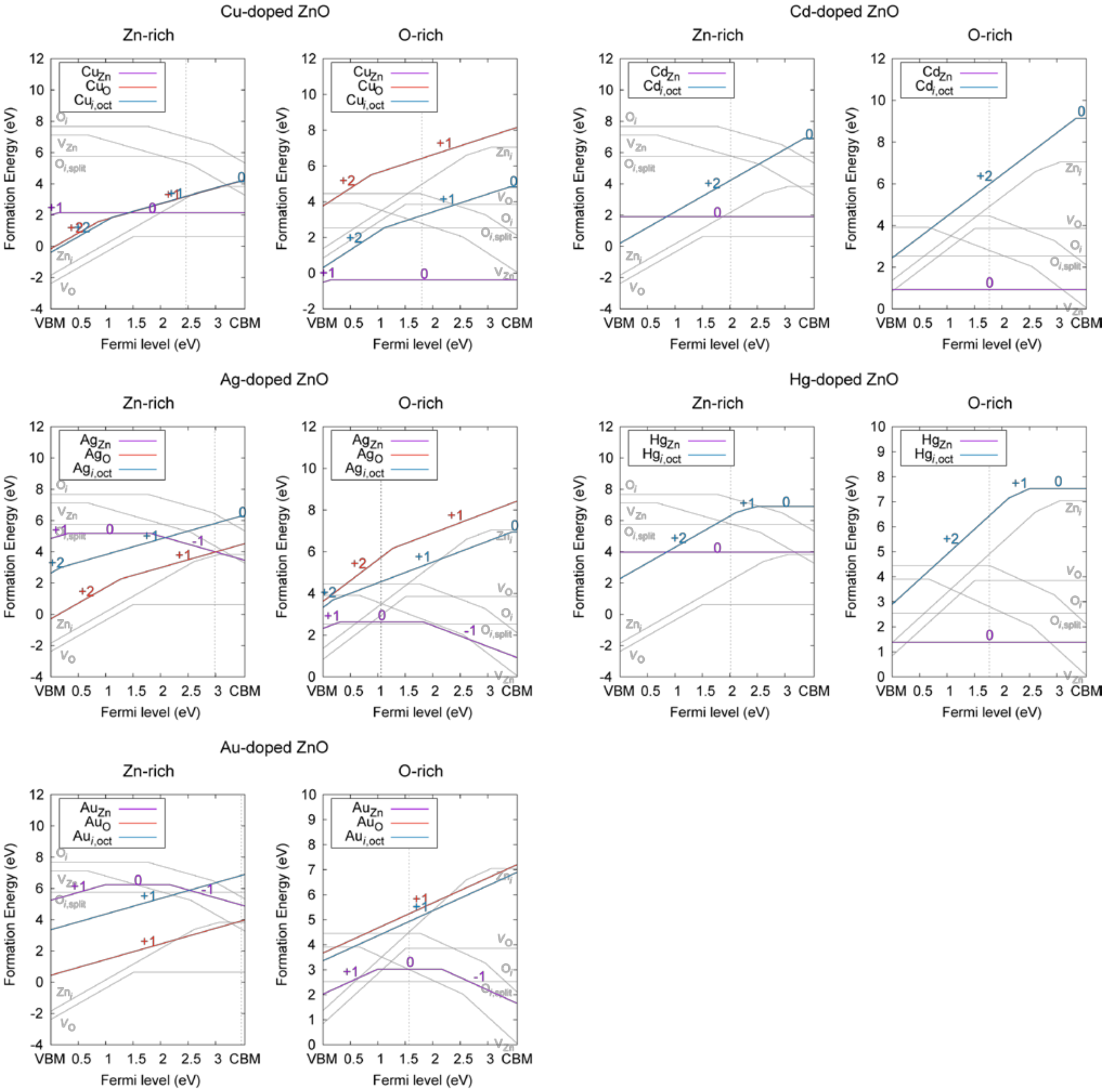

Group 13 elements (IIIA)

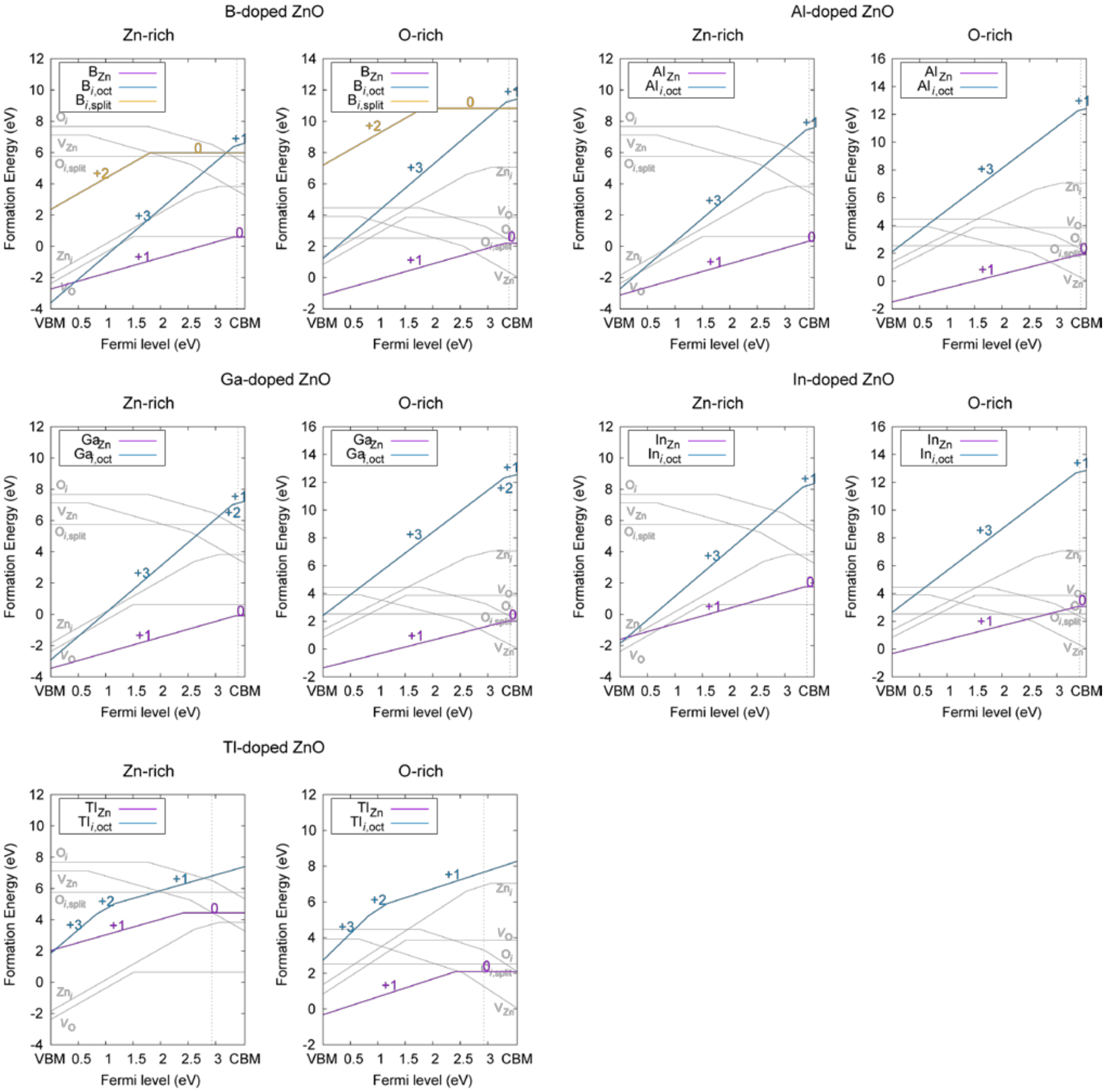

Group 14 elements (IVA)

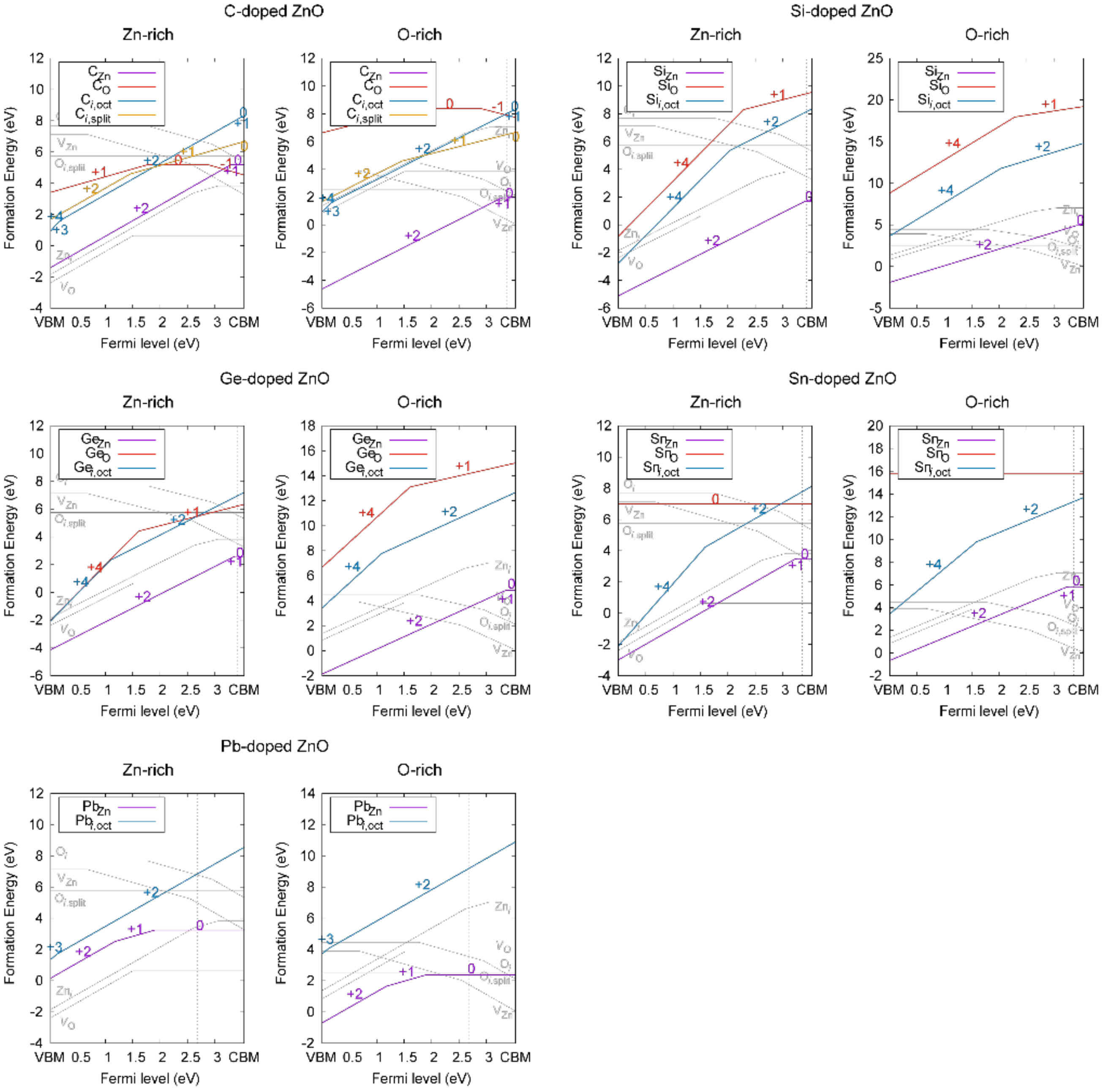

Group 15 elements (VA)

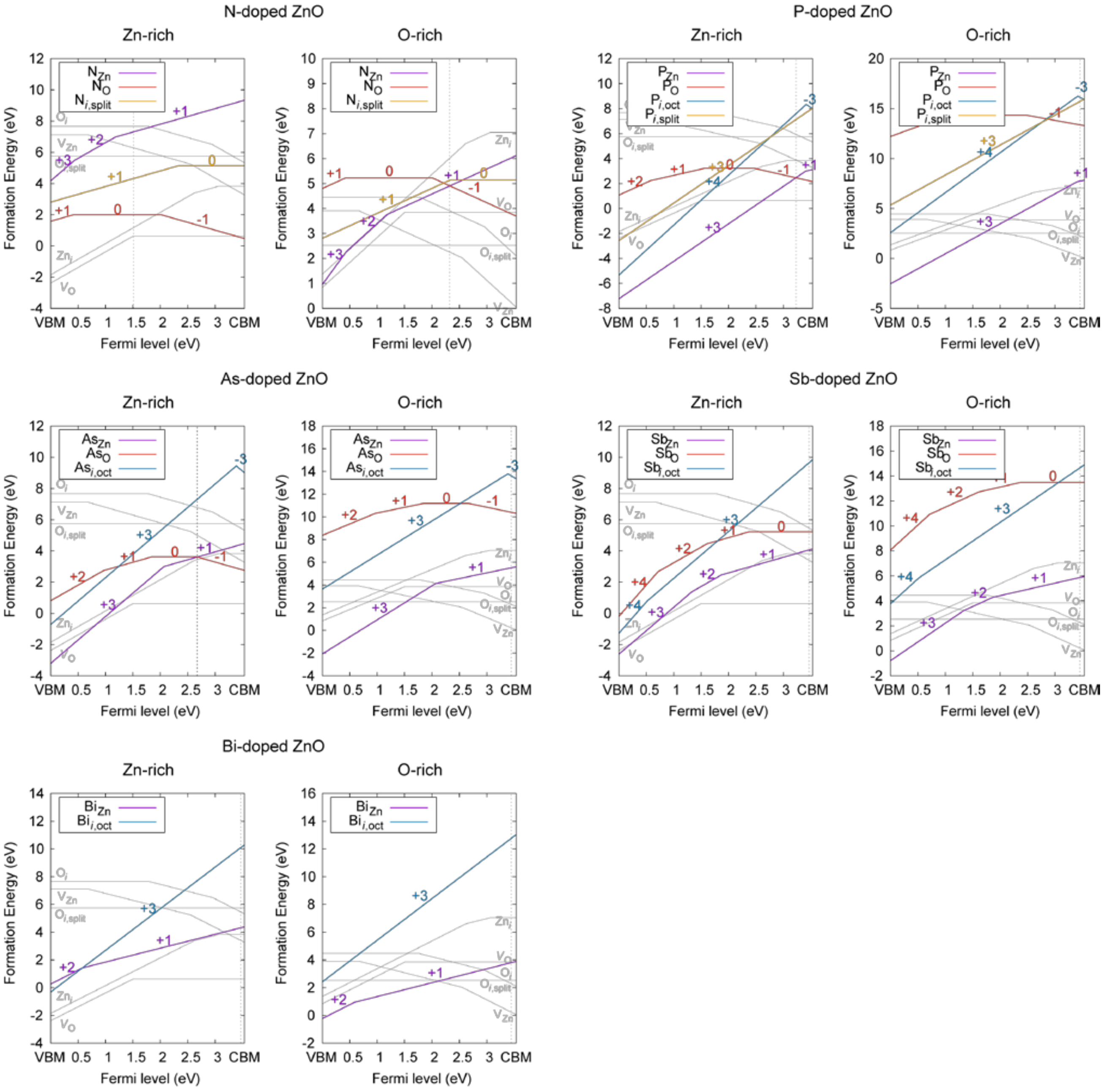

Group 16 elements (VIA)

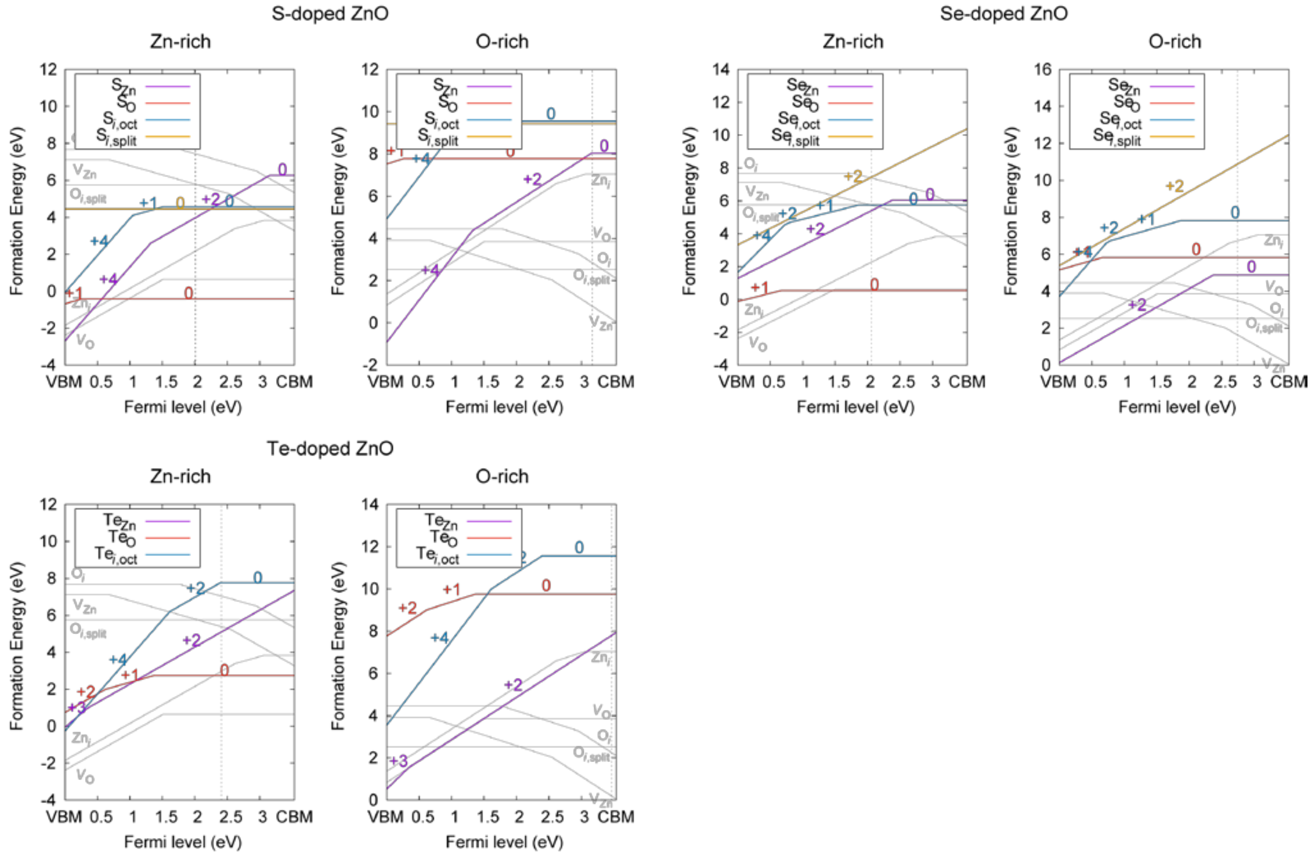

Group 17 elements (VII)

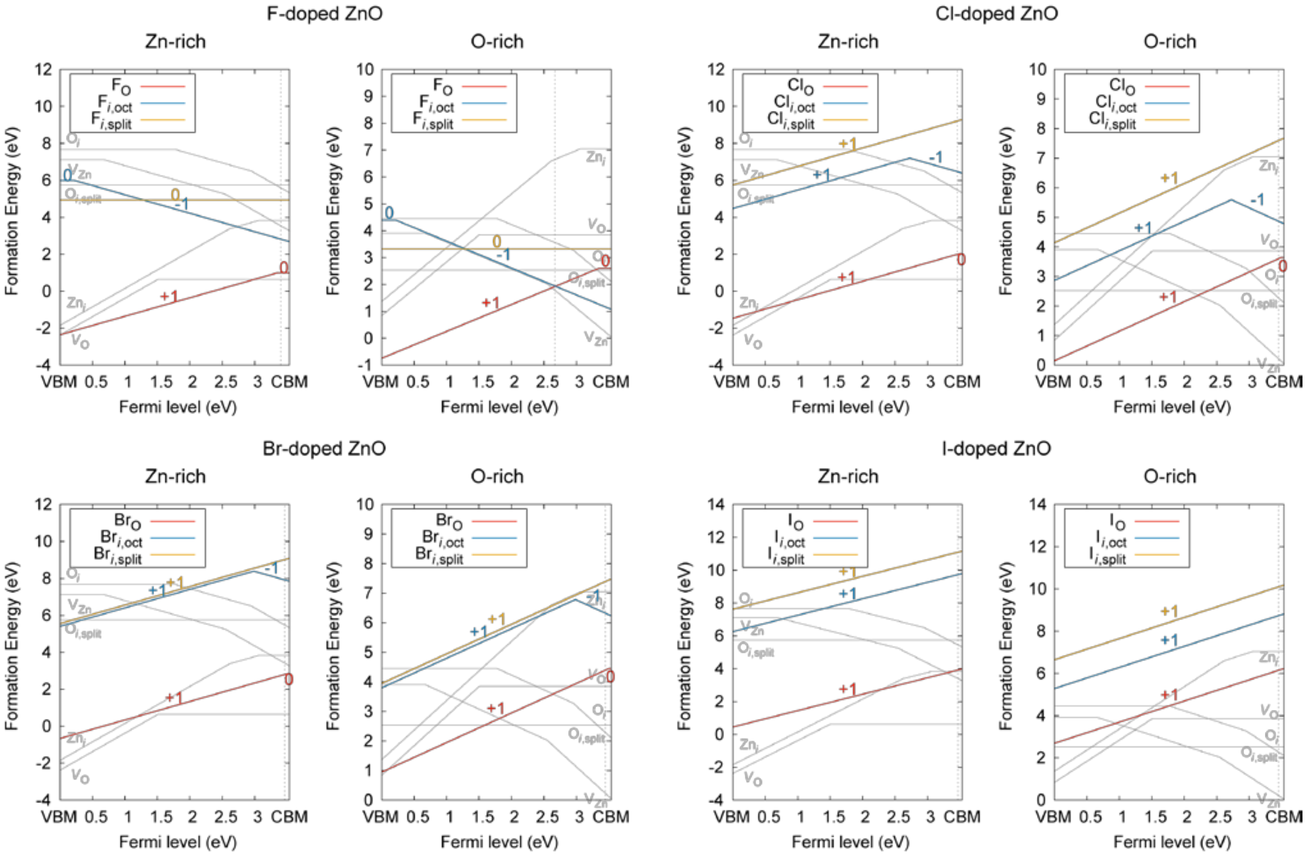

Figure S1. Formation energy plots of 61 elements (arranged by groups in the Periodic Table). For comparison, the formation energies of intrinsic defects are shown in grey.

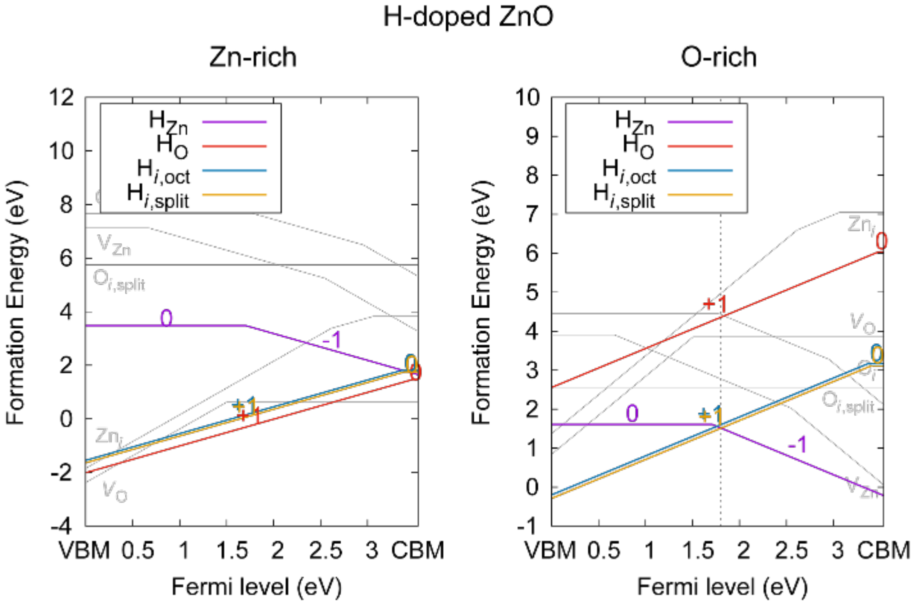

Figure S2. Formation energies of H in ZnO. H_{Zn} is identified as (V_{Zn} + H_i) complex in which the hydrogen atom forms an O-H bond and leaves the zinc site empty.

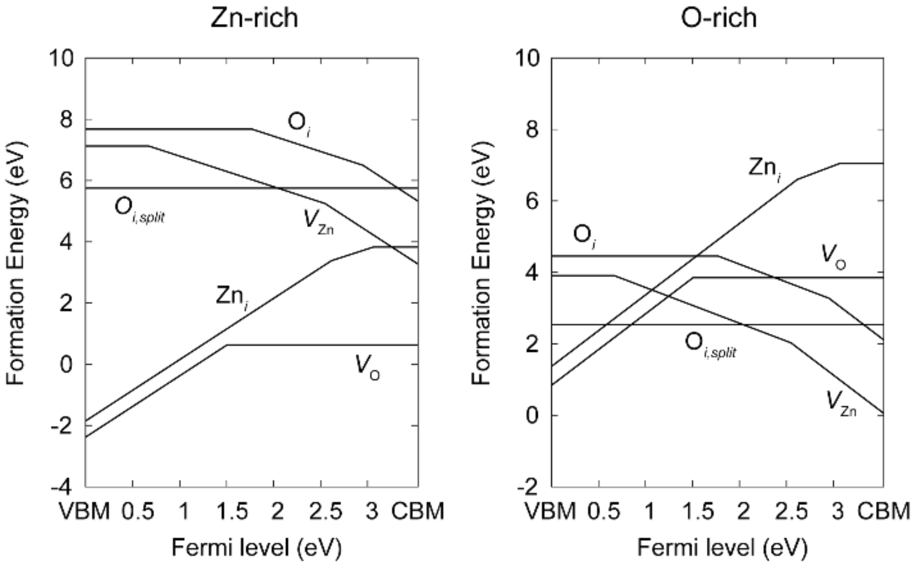

Figure S3. Formation energies of intrinsic defects in ZnO.

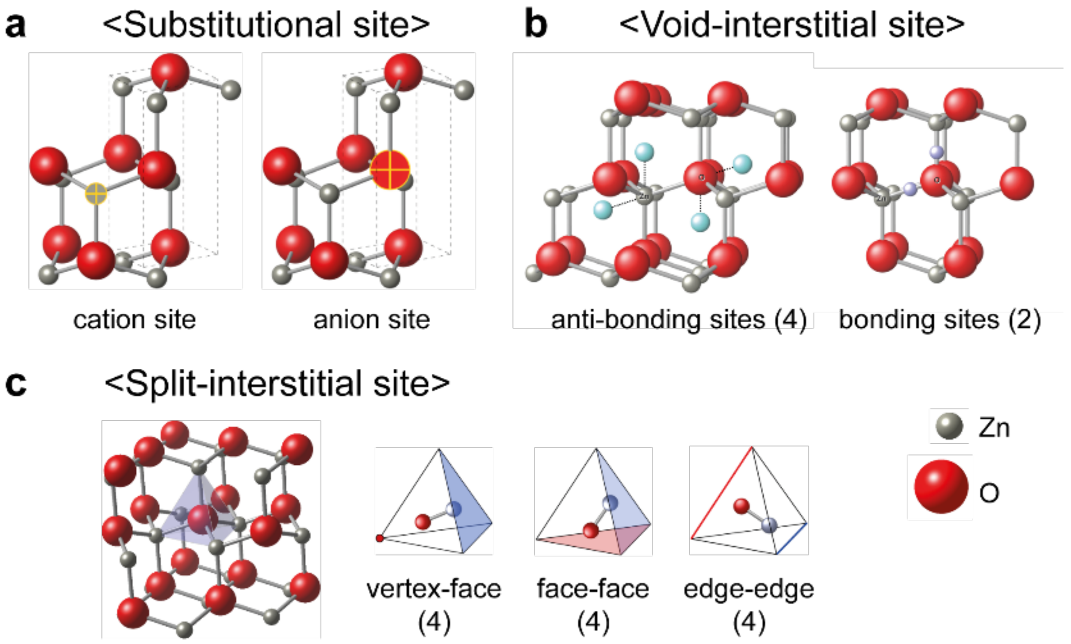

Figure S4. Considered doping sites in ZnO (a) substitutional sites. (b) void-interstitial sites. (c) split-interstitial sites. Number of sites for each type are indicated in the parentheses. In the case of anti-bonding sites for void interstitial, each site lies near octahedral (*i*,oct) or tetrahedral (*i*,tet) sites. It is found that the octahedral site is always more stable than the tetrahedral site for every dopant considered in the present work.

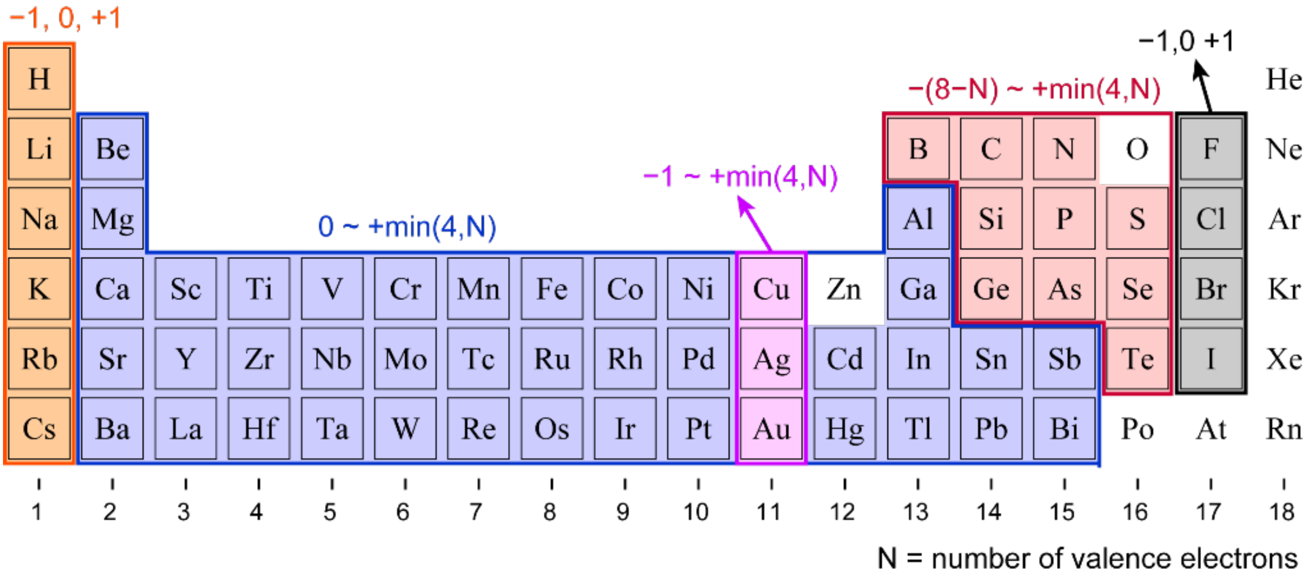

Figure S5. The range of charge state for each dopant.

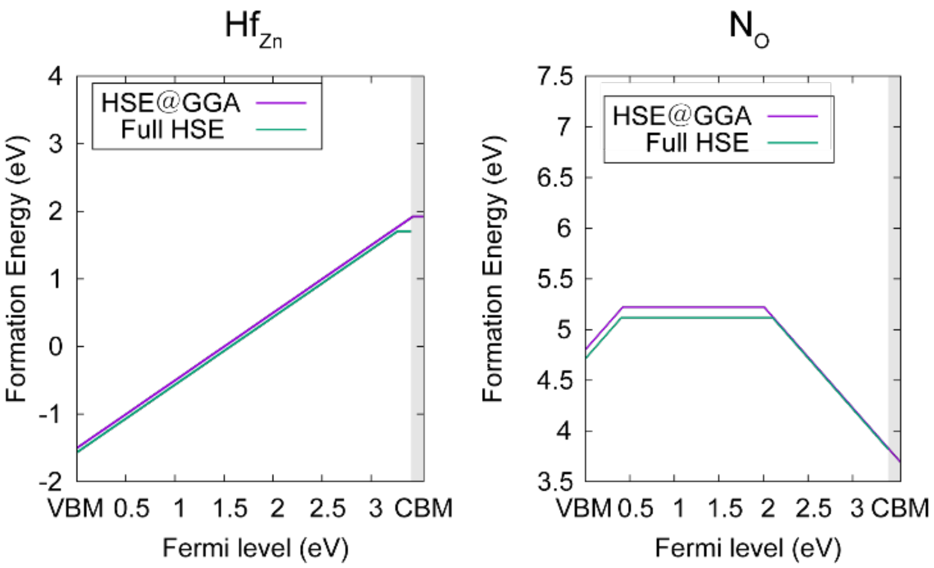

Figure S6. Comparison of formation energies for N_O between one-shot HSE06 calculation based on the GGA+U structure (HSE@GGA) and fully-relaxed HSE06 calculation (Full HSE). Shaded region indicates the difference in the calculated band gap (3.4 eV versus 3.54 eV)

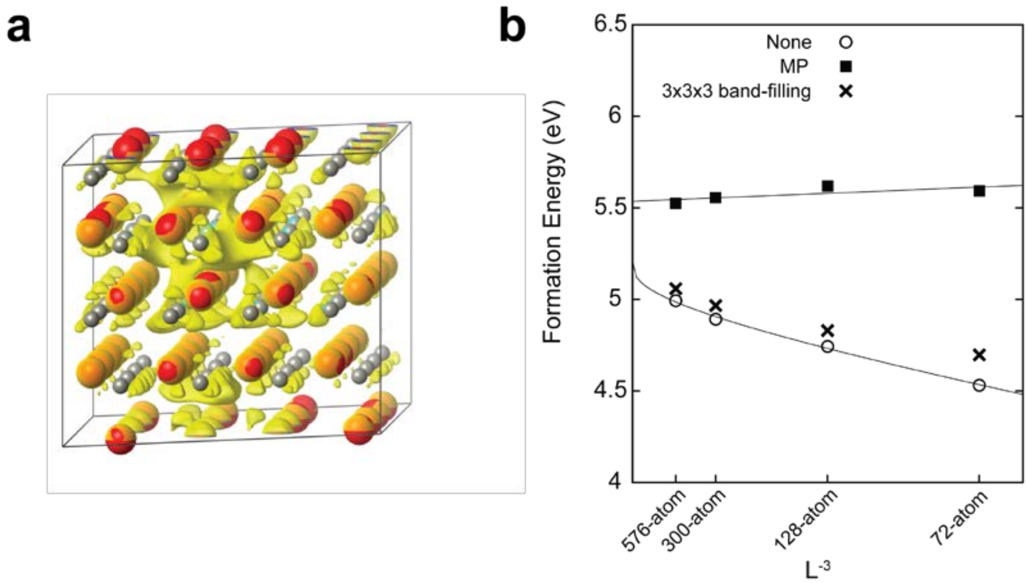

Figure S7. (a) Charge distribution of the defect state in Hf_{Zn}^0. It is rather delocalized over the supercell. (b) The convergence of the formation energy of Hf_{Zn}^0 with and without monopole charge correction (MP) with respect to the inverse of the supercell length. It is clearly seen that MP correction is necessary although the dopant is in the neutral charge state. We also plot the band-filling corrected results using 3×3×3 k-point sampling and it is seen that they are close to Γ-only results.
